# Flamingo-Optimization-Based Deep Convolutional Neural Network for IoT-Based Arrhythmia Classification

**DOI:** 10.3390/s23094353

**Published:** 2023-04-28

**Authors:** Ashwani Kumar, Mohit Kumar, Rajendra Prasad Mahapatra, Pronaya Bhattacharya, Thi-Thu-Huong Le, Sahil Verma, Kavita, Khalid Mohiuddin

**Affiliations:** 1 Department of Computer Science and Engineering, Faculty of Engineering and Technology, SRM Institute of Science and Technology, NCR Campus, Ghaziabad 201204, India; hod.cse.ncr@srmist.edu.in; 2 MIT Art, Design and Technology University, Pune 412201, India; mohit.kumar@mituniversity.edu.in; 3 Department of Computer Science and Engineering, Amity School of Engineering and Technology, Research and Innovation Cell, Amity University, Kolkata 700135, India; pbhattacharya@kol.amity.edu; 4 Blockchain Platform Research Center, Pusan National University, Busan 609735, Republic of Korea; lehuong7885@gmail.com; 5 Faculty of Computer Science and Engineering, Uttaranchal University University, Dehradun 248007, India; sahilverma@ieee.org (S.V.); kavita@ieee.org (K.); 6 Faculty of Information Systems, King Khalid University, Abha 62529, Saudi Arabia

**Keywords:** arrhythmia classification, DCNN, ECG signal, flamingo optimization, IoT nodes

## Abstract

Cardiac arrhythmia is a deadly disease that threatens the lives of millions of people, which shows the need for earlier detection and classification. An abnormal signal in the heart causing arrhythmia can be detected at an earlier stage when the health data from the patient are monitored using IoT technology. Arrhythmias may suddenly lead to death and the classification of arrhythmias is considered a complicated process. In this research, an effective classification model for the classification of heart disease is developed using flamingo optimization. Initially, the ECG signal from the heart is collected and then it is subjected to the preprocessing stage; to detect and control the electrical activity of the heart, the electrocardiogram (ECG) is used. The input signals collected using IoT nodes are collectively presented in the base station for the classification using flamingo-optimization-based deep convolutional networks, which effectively predict the disease. With the aid of communication technologies and the contribution of IoT, medical professionals can easily monitor the health condition of patients. The performance is analyzed in terms of accuracy, sensitivity, and specificity.

## 1. Introduction

An arrhythmia is a cardiac conduction disorder that leads to the death of various individuals and can be noticed using ECG signal recordings. To identify heart diseases such as myocardial infarction, arrhythmia, and ischemic heart disease and to control the regularity of heartbeats, the collected data are used [1]. The irregular functioning of the heartbeat is termed arrhythmia and menaces the life of millions of people around the world. Easy detection of arrhythmia can be performed by keeping track of the heart constantly. However, it is not viable through manual detection. An arrhythmia can be detected by the classification of the heartbeat and the classification will be based on basically five categories: normal, supra ventricular, ventricular ectopic, fusion, and unknown beats, represented by N, S, V, F, and Q, respectively [2]. Cardiac arrhythmias are generally categorized under two categories such as enhanced or abnormal impulse variation and conduction disturbances [3]. For abnormal heart rhythms, cardiovascular disease (CVD) may be the main reason. Earlier, huge methods were used for the monitoring of arrhythmias, which have an ideal effect. Arrhythmias may suddenly lead to death and the classification of arrhythmia is considered a complicated process, so real-time monitoring of arrhythmia is one of the most important processes. For arrhythmia diagnosis [4,5], in order to reduce the work of doctors, an automated detection algorithm through an automatic diagnosis system has been discovered [6]. A homecare management system plays a vital role in the modern world due to the increased number of patients in every hospital. With the aid of communication technologies and the contribution of IoT, the custodian can easily monitor the health condition of the patients. The heart is a sensitive organ in the human body that should be monitored periodically for effective functioning [7].

IoT devices are developed and employed in numerous devices for monitoring, enhancing, and communicating purposes. Furthermore, they provide information at the exact time without the consultant of professionals, which will be more efficient in rural areas. Arrhythmia conditions can be identified by collecting the signals from the individual and evaluating the signal through the analytical tool electrocardiogram (ECG). The important features to be considered for the classification of arrhythmia are as follows: (i) selection of features; (ii) feature extraction; (iii) classification. The challenge to be overcome is that the classification should be performed well even with unbalanced data [8]. In recent years, several optimization algorithms have been used for optimizing the hyperparameters of the classifier. The deep neural network allows for extracting high-level features that are needed for the detection of arrhythmia without human intervention. Wearable clinical devices are invented for the reduction of mortality rates occurring by heart disease arrhythmia by monitoring the heartbeat, blood pressure level, body temperature, and other physical fitness measures.

The main aim is to develop a disease prediction model for the prediction of arrhythmia heart disease. Initially, the data are collected from the victim as an ECG signal and then are preprocessed for the enhancement of the data. The preprocessed data are exposed to the proposed flamingo-based DCNN, where the classification of the disease is performed. The main contribution of the research is as follows:

(1) The flamingo optimization effectively optimizes the hyperparameters of the classifier through the effective handling of the energy associated with bird hunting.
(2) The DCNN model predicts the output with more accuracy and the information is conveyed to the patients in an effective manner for executing the diagnosis.
(3) The data acquisition and classification setup are automatic without any human intervention.
(4) The effective tuning of the classifier helps in the identification of the arrhythmia disease in less time and with faster convergence.

The manuscript is organized as follows: Section 2 enumerates the need for the proposed model by accounting for the advantages and disadvantages of the existing model; Section 3 depicts the system model of the IoT network used in the disease classification; Section 4 gives a detailed version of the proposed flamingo-based DCNN; the obtained results are enumerated and discussed in Section 5; finally, the research is concluded in Section 6.

## 2. Background Study

This section presents a comprehensive discussion of related work on different classification methods. Critical analysis is performed on each article, and if an article is found in more than one repository or database, it will only be taken into account once. The chosen publications provide an overview of many approaches, classification algorithms, and optimization strategies that have been utilized to classify arrhythmias. Jinyuan He et al. [2] introduced a framework for automated arrhythmia detection from IOT-based ECGs that performs multichannel convolutions for capturing both temporal and frequency domain features, which enhances the performance of classification using raw data; however the performance of the solution MCHCNN introduced is not satisfied. Ehsan Moghadas et al. [9] initiated a system for monitoring the health of a patient that attains high accuracy but it has restricted memory that allows only storing the information for a small period of time. R. Lakshmi Devi et al. [4] developed an ECG telemetry system that captures the heart rate efficiently with variable features; the size of the system is very small and it greatly reduces the computational cost. Mohamed Hammad et al. [5] established a deep neural network (DNN) strategy that effectively overcomes the drawback of traditional approaches and it is serviceable in terms of accuracy, sensitivity, positive predictivity, and so on. Areej Almazroa and Hongjian Sun [7] instituted a cardiac arrhythmia monitoring framework that reduces the number of errors that occur in the system, which effectively increases the accuracy, but the classification can be further improved using real data, which enhances the classification. Jilong Wang et al. [10] initiated a human–machine collaborative knowledge representation and it has the capability of hand encoding, but there is the necessity for human intervention. Abol basher et al. [11] established a combined method using a CNN and DNN that automatically detects the disease, but this method uses a small amount of data. Chima S. Eke et al. [12] designed a system relying upon the blood-based biomarkers that detected the arrhythmia disease, but the dimensionality of the data is not reduced. Jianyong Yang et al. [13] initiated an innovative method for the detailed description of the feature extraction and selection methods (WReliefF-GA-SVM), but the noise of the system affects the performance. Yuwen Li et al. [14] extracted the features using the ECG signal provided, but there is a need for improvement in the accuracy. Jagdeep Rahul et al. [15] initiated an improved RR interval-based cardiac arrhythmia classification approach that attains higher accuracy; however, QRS detection is complex in the processed ECG signal. Haoren Wang et al. [6] initiated a dual fully connected neural network model for accurate classification of heartbeats that attains higher sensitivity; on the premise of the high sensitivity, the improvement of the PPVs for abnormal classes is not implemented [16]. Wang Zhiheng et al. proposed a novel swarm intelligence optimization algorithm for flamingo search [17].

The upcoming challenges considered for this research are listed below.

1. Optimizing the hyperparameters using efficient algorithms poses challenges.
2. The most important challenge in homecare systems is accuracy because those systems are dealing with human health which is sensitive and needs high accuracy.
3. A homecare system trusted by health experts should be able to detect abnormalities and make decisions in an accurate way.
4. Long delays should be avoided for improving the efficiency of the models, reducing the delay, and improving the convergence possess challenges.

## 3. System Model of IOT Network

**Figure 1:**
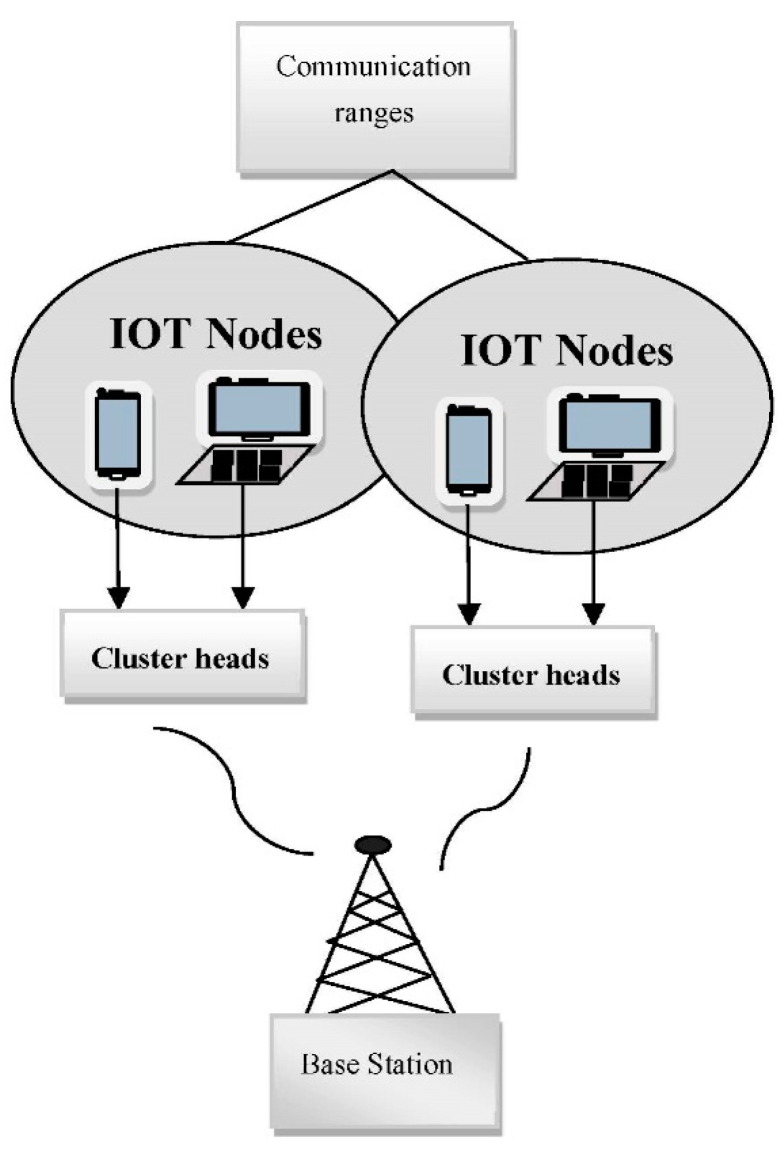
Architecture of an IoT network.

IoT networks make use of various devices, such as mobile phones, smart watches, laptops, sensors, and so on, in order to promote communication and data exchange between them in a certain radio frequency range. The primary contribution of IoT devices is there is no need for human intervention to exchange this information. The IoT network is mainly comprised of three frameworks: the main station, cluster heads, and x, the number of IoT nodes. The IoT network is represented by the factor $V_{m}$, where the nodes x communicate evenly within the distributed range $f_{a}$ and $f_{b}$. Each and every IoT node present in the IoT network receives a unique ID and these nodes are congregated together and form clusters. The clusters formed by the congregation of IoT nodes are transmitted to the respective cluster heads and the cluster heads are represented by the factor $W_{z}$. u is the number of cluster heads in the network and is denoted by (1≤z≤u). The base station (BS) present in the IoT framework receives all the data from the cluster heads. $D_{iz}$ represents the distance between the IoT nodes and the cluster head and the distance between the cluster head and base station is represented by $D_{zj}$. The performance metric that should be considered while designing the IoT network is energy consumption since it is not possible to recharge the nodes present in the network. The energy present at the initial level is represented by $Y_{w}$. While the communication takes place between the cluster nodes and the base station, some energy gets dissipated. The energy dissipation will be determined through the radio electronics and the energy transmitted through the IoT nodes are updated at the base station. The system model of the IoT network architecture is depicted in Figure 1.

## 4. Disease Prediction Using the Flamingo-Optimization-Dependent Deep CNN Classifier

**Figure 2:**
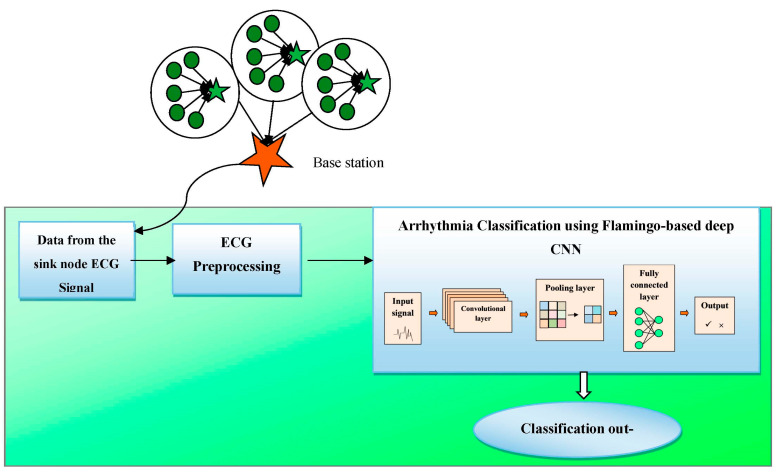
Systematic representation of the arrhythmia prediction model.

This section enumerates the classification of arrhythmia based on the flamingo-optimization-based deep CNN classifier. Initially, the data are obtained from the heart as an ECG signal using the IoT devices that are saved in the base stations. The data from the base station is then preprocessed and the classification is performed using the deep convolutional neural network optimized by flamingo optimization. The proposed flamingo-based-DCNN extracts the features effectively and classifies the patients whether they are affected by arrhythmia or not as well as providing the information at the correct time for providing an effective diagnosis automatically, without the help of a human. For solving classification problems accurately, the DCNN is powerful. For predicting images, the DCNN has the capability to provide the highest accuracy among other classifiers. The DCNN is fast to implement and when working with images DCNN has more advantages. The proposed system model for the detection of arrhythmia is shown in Figure 2.

### 4.1. Preprocessing

The input ECG data are used for the detection of the arrhythmia disease and as an initial step, the data are preprocessed utilizing the standard scalar transform method, where the normalization of the data and removal of irrelevant noise takes place.

### 4.2. Proposed Flamingo-Based Optimization for Arrhythmia Classification

**Figure 3:**
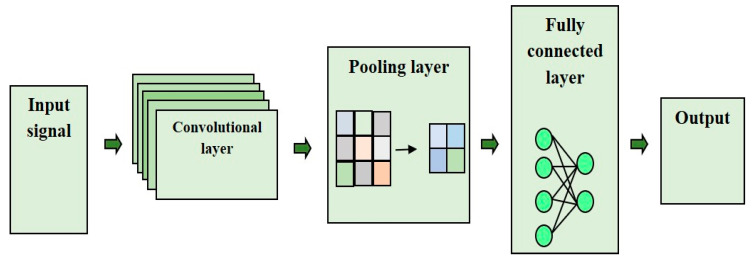
Architecture of the deep CNN.

Deep CNN classifiers are considered to be very efficient because they have the capability to reduce the number of parameters without compromising the quality of the data. The deep CNN classifier also provides faster convergence than other classifiers. In the proposed model, the disease classification is performed using the flamingo-optimization-based deep CNN, which directly optimizes the parameters from the provided data. The DCNN network effectively extracts the confined features from the input, which yields better classification performance. The deep CNN network trains the data in a well-defined manner and classifies the disease by utilizing the layers as follows: convolutional layer, pooling layer, and fully connected layer. The architecture of the DCNN is depicted in Figure 3. The main advantage of DCNN compared to its predecessors is that it automatically detects important features without any human supervision. For effective training of the classifier, Flamingo optimization is used as an optimizer to effectively learn from the input data and reveal the effective classification performance.

Convolutional layer: The convolutional layers have the ability to learn the features of a large number of filters and filter the most necessary and highly effective features needed for the classification. The filters are applied to initiate a feature map that effectively summarizes the important features. The convolution layer extracts the features relevant to the arrhythmia classification.

Pooling layer: The dimensions of the features obtained from the convolutional layer are reduced by the down-sampling method in the pooling layer. Downsampling is performed for the reduction of dimensionality and helps in improving accuracy.

Fully connected layer: The classification of the disease is enhanced by feed-forwarding the inputs from the initial layers to the final layers. The high-level features obtained from the convolutional layers are flattened and the weight, bias, and neurons will be present in the fully connected layer and form an intermediate between the input and output layers. The fully connected layer provides the output as to whether the individual is affected by the disease or not.

#### Flamingo Optimization for Tuning the Hyperparameters of the Classifier

To determine the global optimal solution in the given search space, flamingo optimization is used, which replaces the standard Adam optimizer in the deep CNN. The flamingo optimization has high application capability, high foraging capability, and high global search characteristics, making the optimization more suitable for tuning the classifier. The flamingo optimization is enabled in the deep CNN classifier for deriving the optimal solution by tuning the hyperparameters. The weights and bias in the deep CNN classifier act as the learnable parameters, hence they are optimally tuned for effective classification of the disease.

Inspiration: Flamingos are gregarious birds that live in flocks and exhibit the characteristics of scavenging and emigrating behavior, which greatly helps in the optimization process. Initially, the foraging characteristics of the flamingos are categorized into sociable, bill-scanning, and claw locomotive behavior. Using this behavior, foraging is performed and then global optimization is performed under limited resources. The characteristics of flamingos are incorporated in the deep CNN classifier for the efficient classification of arrhythmia disease. The internal hyperparameters of the deep CNN classifier are tuned optimally to derive the best solution and the steps involved in the flamingo optimization are mathematically expressed in the sections below.

**(i) Scavenging behavior**

Sociable behavior: Initially the flamingo that discovers the food will communicate to the other flamingos and made them change their position corresponding to the location of the food. If the flamingo tries to find the optimal solution, which is where there is an abundance of food, we mathematically expressed the abundance of food in $k^{th}$ dimension as $yd_{k}$.

**(ii) Fitness function**

The fitness function of each flamingo is evaluated and the best flamingo is considered the fittest solution that provides the optimal candidate solution. The fitness function is given by,

(1) $$f\left(x\right)=100\left(x_{1}^{2}-x_{2}\right){\;}^{2}+\left(1-x_{1}\right){\;}^{2}$$

The solution corresponding to the maximal value of the fitness is declared as the global solution to update the classifier.

**(iii) Bill-scanning behavior**

A flamingo searches for its food by immersing its head in the water and when the food gets grabbed it swallows the food in an upside-down manner where the filtration of excess water and waste materials takes place. When there is an abundance of food, the flamingos tilt their head and scan more carefully, and depending on the situation, the scanning radius varies. We assign the position of the $l^{th}$ flamingo in the $k^{th}$ dimension as $y_{lk}$. During the exchange of information, random errors can occur which are conquered by the implementation of standard normal distribution, even though there is a probability of occurring minor errors. The maximum distance covered by the flamingos is mathematically expressed as

(2) $$A_{1}=M_{1}\times yd_{k}+\lambda_{1}+y_{lk}$$

Initially, the assumption is made that the scanning is performed at its maximum distance and $M_{1}$ is a random number that follows a uniform distribution, the variation in the scanning range of the flamingos is represented by

(3) $$A_{2}=M_{2}\times |M_{1}\times yd_{k}+\lambda_{1}+y_{lk}|$$

$M_{2}$ is a random value that follows a standard uniform distribution, $\lambda_{1}$ and $\lambda_{2}$ are random numbers in the range [−1,1].

**(iv) Claw locomotive behavior**

The claws of the flamingos move toward the location where there is an abundance of food. The location where there is a high amount of food is denoted as $yd_{k}$ and the distance covered by the flamingos is $\lambda_{1}\times yd_{k}$ which increases the search area. The movement of the steps of searching for food in the $n^{th}$ iteration is given by

(4) $$s_{lk}^{n}=\lambda_{1}\times ys_{k}^{n}+M_{2}\times |M_{1}\times yd_{k}+\lambda_{1}+y_{lk}|$$

The location of the flamingo is updated with varying locations and is mathematically expressed as

(5) ylkn+1=(ylkn+λ1×yskn+M2×|M1×ydk+λ1+ylk|)R

Here, $y_{lk}^{n+1}$ represents the $l^{th}$ flamingo in the $k^{th}$ dimension in the (n+1) iteration and $y_{lk}^{n}$ is the $l^{th}$ flamingo in the $k^{th}$ dimension in the $n^{th}$ iteration. $ys_{k}^{n}$ is the current best solution in the $n^{th}$ iteration. R=R(q) is the diffusion factor that assigns a random number that follows the chi-square distribution of q degrees of freedom. The diffusion factor is utilized to increase the range of the searching area which improves the global optimal solution. $M_{1}=V\left(0,1\right)$ and $M_{2}=V\left(0,1\right)$ are random factors that follow the standard normal distribution.

**(v) Emigrating behavior**

The emigrating flamingo is considered the fittest and the fitness value is assigned to the flamingos based on the capability of emigrating. When the food present in the area is reduced, the flamingos shift toward a new position in search of food where there is abundance. The emigrating behavior of the flamingo is mathematically represented as follows

(6) $$y_{lk}^{n+1}=y_{lk}^{n}+\sigma \left(ys_{k}^{n}-y_{lk}^{n}\right)$$

where $y_{lk}^{n+1}$ represents the position of the $l^{th}$ flamingo in the $k^{th}$ dimension in the n+1 iteration. Similarly, $y_{lk}^{n}$ represents the position of the $l^{th}$ flamingo in the $k^{th}$ dimension in the $n^{th}$ iteration. $ys_{k}^{n}$ is the best fitness solution for the population. σ=V(0,q) is a Gaussian random number with q degrees of freedom, which is used to simulate individual behavior and large search space for obtaining the optimal solution. Algorithm 1 shows the pseudocode of the flamingo optimization. Hence the optimal solution is updated in the deep CNN.

**(vi) Termination**

After reaching the maximum number of iterations, the optimal solution is determined and the process is terminated.


**Algorithm 1:** Pseudocode for flamingo optimization.
**1**
 **Initialization**
2 **Input:**
$yd_{k}$
3 **Output:**
$y_{lk}^{n+1}$
4 **While**
$\left(n<n_{\max}\right)$   # $n_{\max}$ is the maximal iterations5 **Scavenging behavior**
6   **If (Sociable behavior)**
7     **Initialize:**
$yd_{k}$
8   **For Bill scanning behavior do**
9     **Maximum distance**
10      $A_{1}=M_{1}\times yd_{k}+\lambda_{1}+y_{lk}$
11     **Varying scanning range**
12      $A_{2}=M_{2}\times |M_{1}\times yd_{k}+\lambda_{1}+y_{lk}|$
13     **Claw locomotive behavior**
14   $s_{lk}^{n}=\lambda_{1}\times ys_{k}^{n}+M_{2}\times |M_{1}\times yd_{k}+\lambda_{1}+y_{lk}|$
15     **Position update**   ylkn+1=(ylkn+λ1×yskn+M2×|M1×ydk+λ1+ylk|)R16 **Emigrating behavior**
17   $y_{lk}^{n+1}=y_{lk}^{n}+\sigma \left(ys_{k}^{n}-y_{lk}^{n}\right)$
18 **Check the stopping condition**
19 **End**



## 5. Results and Discussion

Arrhythmia diseases are detected using the deep learning network and the results obtained are discussed in detail in the section below.

### 5.1. Dataset Description

The dataset used for the prediction of arrhythmia disease is the MIT-BIH dataset, with 4000 recordings from the clinical lab, where more than half of the data have been acquired from in-patients. The recordings last about 30 min and include information on complex ventricular, junctional, and supraventricular arrhythmias and conduction abnormalities using the rhythm features and QRS alterations. The data are from men and women aged above 22 years. The dataset used for the prediction of arrhythmia disease is the MIT-BIH dataset and the MIT-BIH arrhythmia database consists of a two-channel ambulatory ECG signal collected from 47 objects in the BIH Arrhythmia Laboratory (https://physionet.org/content/mitdb/1.0.0/ (accessed on 2 February 2023). 

### 5.2. Experimental Setup

The arrhythmia disease classification is performed using Python and the system configuration is enlisted as follows: Python 3.7.6 running in the platform pycharm 2020-community edition in the Windows 10 operating system. The experimentation is carried out using the MIT-BIH database.

### 5.3. Performance Metrics

The performance metrics are used to evaluate the significance of the proposed disease prediction model.

Accuracy: The percentage of the correctly categorized instances while performing disease prediction is called accuracy and is given by

(7) $$accu=\frac{True_{pos}+True_{neg}}{True_{pos}+True_{neg}+False_{pos}+False_{neg}}$$

Sensitivity: The percentage of the actual positive values which are correctly identified during arrhythmia prediction is called sensitivity and is given by

(8) $$sens=\frac{True_{pos}}{True_{pos}+False_{neg}}$$

Specificity: The percentage of the measure of actual negative values that are correctly identified during the arrhythmia prediction is called specificity and is given by

(9) $$Spec=\frac{True_{neg}}{True_{neg}+False_{pos}}$$

### 5.4. Evaluation Based on Performance for Arrhythmia Prediction Model

**Figure 4:**
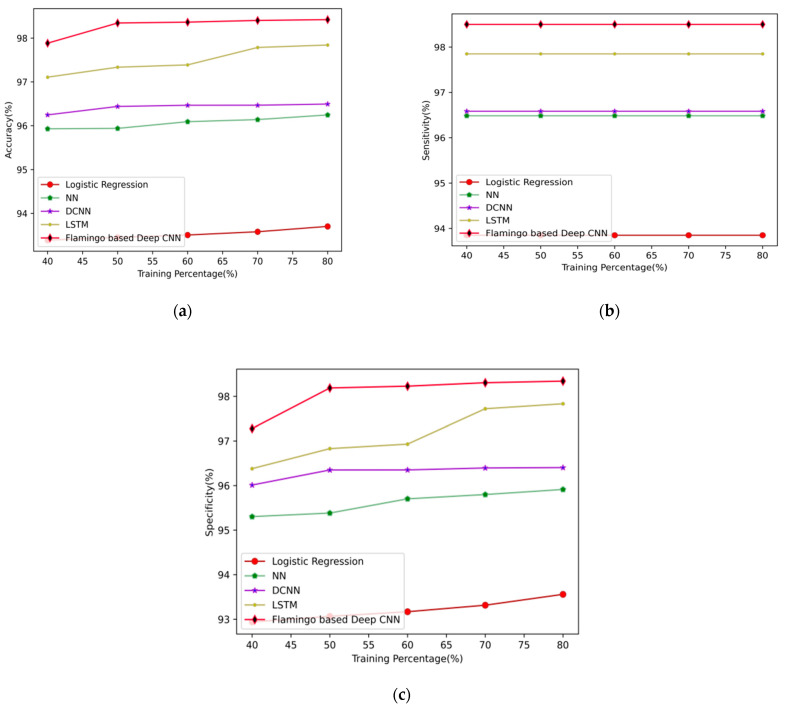
Comparative analysis of arrhythmia disease prediction using (**a**) accuracy, (**b**) sensitivity, and (**c**) specificity.

The performance of the proposed flamingo-based DCNN for the prediction of arrhythmia disease at varying epochs is shown in Figure 4. The accuracy rates of the proposed flamingo-based DCNN for the epoch value 100 using 80% of the training data are 97.612%, 97.774%, 97.808%, 97.821%, and 97.836%, respectively. Similarly, the sensitivity rates at the epoch rate of 100 using 80% of the training data for the proposed flamingo-based DCNN method are 97.850%, 97.850%, 97.850%, 97.850%, and 97.850%, respectively. Finally, the specificity rates at epoch 100 are 97.472%, 97.698%, 97.767%, 97.793%, and 97.823%, respectively, for the proposed flamingo-based DCNN method. It shows that when the epoch value is high, the system prediction is more accurate and precise.

### 5.5. Comparative Evaluation

This section evaluates the improvement in the performance of the proposed model and is enumerated in a distinct manner. The comparative analysis is performed using the metrics of accuracy, sensitivity, and specificity is discussed below in detail.

#### Comparative Methods

The methods used for the comparison are logistic regression [18], neural network (NN) [19], deep convolutional neural network (DCNN) [20], and long short-term memory (LSTM) [21], and they are compared with the proposed flamingo-based DCNN.

### 5.6. Comparative Analysis for Arrhythmia Prediction Model

**Figure 5:**
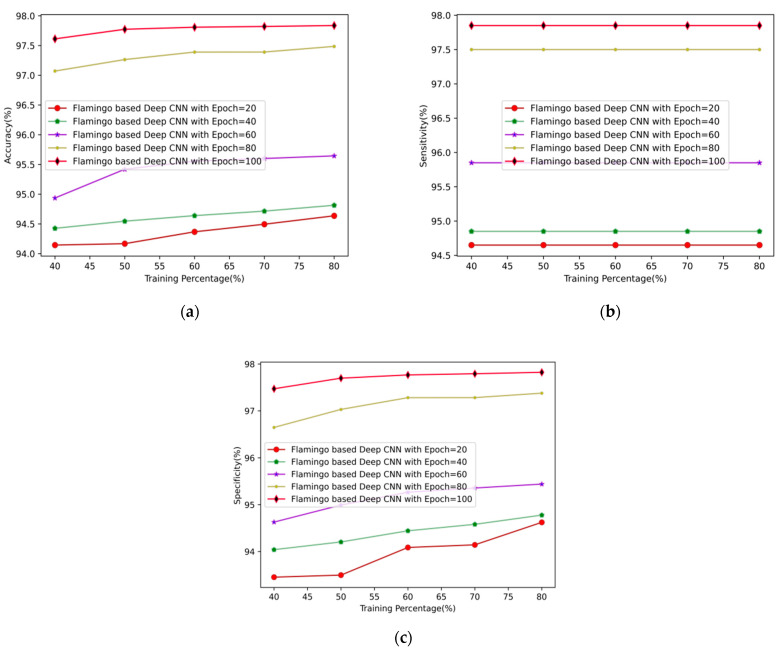
Performance analysis of arrhythmia disease prediction at varying epochs using (**a**) accuracy, (**b**) sensitivity, and (**c**) specificity.

**Figure 6:**
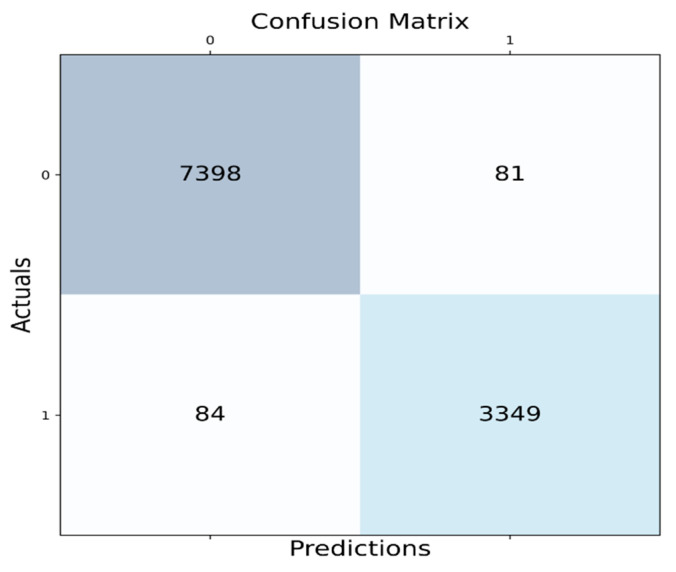
Confusion matrix.

Initially, the accuracy rates of the methods logistic regression, neural network, deep convolutional neural network, long short-term memory, and proposed flamingo-based DCNN are measured for 80% of the training data and the values obtained are 97.883%, 98.343%, 98.363%, 98.402%, and 98.420%, respectively. Similarly, the sensitivity rates of logistic regression, neural network, deep convolutional neural network, long short-term memory, and the proposed flamingo-based DCNN are 98.500%, 98.500%, 98.500%, 98.500%, and 98.500%, respectively, for a training percentage 80%. Finally, the specificity rates of logistic regression, neural network, deep convolutional neural network, long short-term memory, and the proposed flamingo-based DCNN are measured and listed as 97.279%, 98.187%, 98.227%, 98.304%, and 98.341%, respectively, for the training percentage of 80. From this observation, it can be proved that the proposed method is more efficient, and the observations are depicted in Figure 5. A confusion matrix is shown in Figure 6.

### 5.7. Comparative Discussion

The comparative discussion is performed to discuss the superiority over the existing methods by the proposed flamingo-based deep CNN model. The values obtained by the proposed model at various training percentages are interpreted in Table 1. The observation shows that when the training percentage is high, the output values are also high due to the fact that when there is information about the features that are sufficient for the classification, the metrics values also get improved. The improvement rate obtained in the classification is due to the optimization enabled in the classifier, which helped in calibrating the parameters inside the deep CNN classifier and improved the performance of arrhythmia classification.

## 6. Conclusions

Arrhythmia is a deadly disease that can affect the lives of many people. In order to prevent the destruction caused by this disease, it should be detected at an earlier stage so that the diagnosis starts at an earlier stage. In the proposed flamingo-based DCNN model, the arrhythmia disease is predicted with higher accuracy, sensitivity, and specificity rate compared to traditional methods. Initially, the data present in the IoT nodes as an ECG signal are collected, preprocessed, and then predicted using the proposed DCNN model, where the classifier network is optimized using flamingo optimization. Using the bird hunting strategy, the flamingo optimization effectively optimizes the classifiers’ hyperparameters in an effective manner. The DCNN model predicts the output with more accuracy and when executing the diagnosis, the information is transferred to the patients in an effective manner. The accuracy acquired by the proposed method is 98.5%, which shows higher performance compared to existing methods.

## Figures and Tables

**Table 1:** Comparative discussion of the arrhythmia classification model.

Metrics	Methods	Training Percentage (%)				
		40	50	60	70	80
Accuracy (%)	Logistic regression	93.39	93.45	93.51	93.58	93.70
	NN	95.93	95.94	96.09	96.14	96.25
	DCNN	96.25	96.44	96.47	96.47	96.49
	LSTM	97.11	97.33	97.39	97.79	97.84
	ABC + ACO [22]	-	-	-	-	90.83
	FABC + EACO [22]	-	-	-	-	92.74
	Threshold + ACO [22]	-	-	-	-	94.75
	FABC + FBeeBAT [22]	-	-	-	-	96.67
	MRFO [23]	-	-	-	-	98.26
	DA [23]	-	-	-	-	97.98
	HHO [23]	-	-	-	-	97.96
	GOA [23]	-	-	-	-	97.57
	GWO [23]	-	-	-	-	97.89
	SSA [23]	-	-	-	-	98.07
	PSO [23]	-	-	-	-	97.65
	ALO [23]	-	-	-	-	98.11
	Proposed	97.88	98.34	98.36	98.40	98.42
Sensitivity (%)	Logistic regression	93.85	93.85	93.85	93.85	93.85
	NN	96.48	96.48	96.48	96.48	96.48
	DCNN	96.58	96.58	96.58	96.58	96.58
	LSTM	97.85	97.85	97.85	97.85	97.85
	Proposed	98.50	98.50	98.50	98.50	98.50
Specificity (%)	Logistic regression	92.94	93.06	93.17	93.31	93.56
	NN	95.30	95.38	95.70	95.80	95.91
	DCNN	96.01	96.35	96.35	96.39	96.40
	LSTM	96.38	96.83	96.93	97.72	97.83
	Proposed	97.28	98.19	98.23	98.30	98.34

## Data Availability

Data will be provided upon request.
